# Noise is an underrecognized problem in medical decision making and is known by other names: a scoping review

**DOI:** 10.1186/s12911-025-02905-z

**Published:** 2025-02-17

**Authors:** Kayla V. Dlugos, Mjaye Mazwi, Robert Lao, Osami Honjo

**Affiliations:** 1 Institute of Medical Science, University of Toronto, Toronto, Canada; 2 Division of Cardiovascular Surgery, The Labatt Family Heart Centre, The Hospital for Sick Children, Toronto, Canada; 3 Department of Surgery, University of Toronto, Toronto, Canada; 4 Division of Cardiac Critical Care, Seattle Children’s Hospital, Seattle, WA USA; 5 NorCliffe Foundation Center for Integrative Research, Seattle Children’s Research Institute, Washington, USA; 6 Division of Cardiovascular Surgery, Watson Family Chair in Cardiovascular Science, Hospital for Sick Children, University of Toronto, Toronto, Canada

**Keywords:** Noise, Random variability, Medical decision making

## Abstract

Unwanted random variability in day-to-day decision making referred to as ‘noise’ is associated with unhelpful variation that affects both the reproducibility and quality of decision making. Although this is described in other fields, the prevalence of noise in medical decision making and its effects on patient outcomes and the process and efficiency of care have not been reported and are unknown. This review sought to explore noise as a feature of medical decision making, as well as explore potential sources of noise in this setting. The search generated 2,082 results. Analysis of 14 studies included in the review (11 PubMed, 3 reference mining) suggests noise is a driver of unhelpful practice variation and may have important effects on care efficiency and reproducibility. 7 of the 14 studies demonstrated pattern noise, 3 demonstrated occasion noise, and 5 demonstrated stable pattern noise. The decision making in 8 studies demonstrated level noise, and lastly the decision making in 4 of the studies demonstrated system noise, a combination of both pattern and level noise. Additional study is required to ascertain how to measure and mitigate noise in medical decision making, as well as better understand the sources of noise present. Clinical trial number not applicable.

## Introduction

Unwanted random variability of judgment, referred to as ‘noise’, occurs in decision making and is seen across many disciplines [13]. In the broadest sense, noise can be broken down into two subgroups including *intra-rater reliability* (the consistency of an individual’s decisions over time) and *inter-rater reliability* (the consistency or agreement among individuals’ decisions over time) [13]. Some examples include the valuing of stocks, the sentencing of criminals, or in the evaluation of the collective performance of a company’s employees. Many decisions made in medical settings are matters of judgment. ‘Noise’ may be an important problem in medical decision making which includes establishing diagnoses, assessing conditions, or deciding upon management options among others, but this has yet to be systematically evaluated [8]. A judgment assigns a score to an object of focus, integrating diverse pieces of information into an overall assessment [13]. Some judgements are *predictive*, meaning we attempt to come close to a true value, and while some predictive judgements are verifiable in medicine, many are unverifiable [13] with a common example being a judgment that a given therapeutic strategy is likely to be more effective than the alternative with no ability to test the counterfactual outcome. Adding further complexity, many judgements are not *predictive* rather they are *evaluative*, such as in cases where something cannot be compared to a true value [13]. An example of an evaluative judgment can be seen in an example where an individual is to sentence a felon. This is not a prediction rather it is an evaluative judgment that seeks to match the sentence to the severity of the crime [13]. When making a judgment not only are individuals aiming at a hypothetical target but they also assume that others around them are also coming to a similar judgment [13].

When errors in judgments trend towards one direction; this *average error* is referred to as bias [13]. Errors however remain after bias is removed in the form of unwanted divergence of judgements; this divergence is *noise* [13]. While it has been demonstrated that removing bias will help to improve accuracy of judgements, noise is less well studied and it is not as clearly demonstrated how removing noise will lead to improvements [13].

**Fig. 1:**
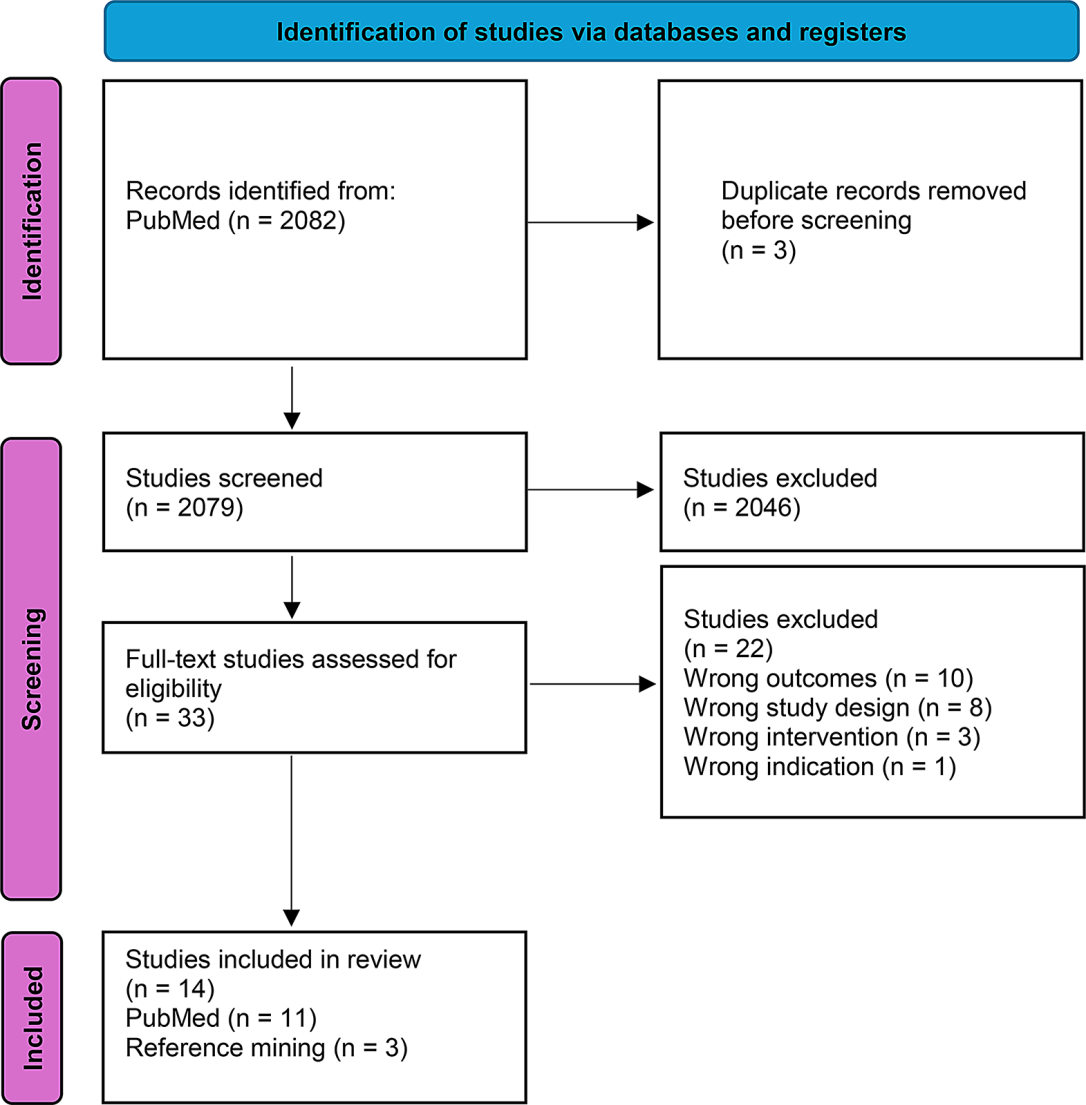
Types of noise categorized by Kahneman et al., [13]. System noise which can be subdivided into pattern and level noise. Pattern noise can be further subdivided into stable pattern and occasion noise

Noise can manifest in clinical practice in a variety of ways. *System noise* is observed when interchangeable professionals make decisions [13]. System noise is broken down into *level* noise and *pattern* noise. Level noise is the variability of the average level of different professionals’ judgements [16] or in other words can be defined as inter-rater reliability. Pattern noise, which can be defined as intra-rater reliability, is the variability of a single professional’s decisions and can be further broken down into *occasion noise* and *stable pattern noise*. See Fig. 1. Occasion noise results from a variety of factors that depend on when a decision is made such as stress, fatigue, hunger, and workload among others Stable pattern noise refers to the difference between two individuals’ judgements with the same set of data that has become stable over time [16].

Consider the following example where two male patients are sitting in the waiting area of a cardiology clinic. After some discussion they realize they are both attending the clinic for the same problem - chest pain during their workday. They meet following their appointments, only to find out that one was told he is simply experiencing pain from a muscle strain while the other was referred for an angiogram [16]. When medical professionals make different decisions despite being faced with the same information this can be termed noise - unwanted, random variability in decision making [16]. If the patients were seen by different medical professionals, then the difference in behavior between the two could be considered an example of level noise, the variability of the average level of different professional’s judgments across different sets of data [16]. If the patients were evaluated by the same doctor, this could be an example of pattern noise, referring to the variability of a single professional’s decisions [16]. Further breaking down pattern noise, stable pattern noise is the difference between two individuals’ judgments with the same set of data [16]. In this case, the doctor could have scheduled the first patient for an angiogram because he works as a farmer and the doctor always refers farmers with chest pain for angiography due to previously having missed an important diagnosis on a patient who was a farmer [16]. On the other hand if the doctor was running late at the end of clinic when he saw the second patient, perhaps he told him it was simply a muscle strain that seems to be fine due to time constraints and needing to rush - this would be an example of occasion noise [16].

It is important to note the potential relation between the reliability of a measure and the limits on validity due to measure unreliability. Validity and reliability measurement issues do exist when it comes to assessing physicians’ performance [14]. The selection of behaviors and results that serve as performance dimensions, as well as the determination of types of raters (by occupational status or role in the healthcare delivery system) who can most accurately assess the performance of physicians are measurements that are not exempt from flaws [14]. Studies of validity of various performance dimensions (such as medical decision making in this case) suggest that a combination of behaviors and results are needed to be able to assess performance accurately and thoroughly [23].

Noise likely plays a large role in error, contradicting a commonly held belief that random errors do not matter, under the assumption that they “cancel out” [13]. An example is widely varying rates of referral for coronary angiography indicate that some patients are not receiving the appropriate investigation they need, while some patients are being over investigated [2]. The noise in this example suggests that some values are too “high” and some values are too “low” and while they may result in an overall average that appears to be appropriate, the reality remains that some patients are over investigated while others are being under investigated, resulting in systemic unfairness and highly variable patterns of care and outcomes at the level of the individual patient [16]. The care that patients receive should be consistent regardless of who they see or when they are seen [16]. It is therefore fitting to say that noise likely represents an important understudied problem in medical decision making and mitigation strategies should be explored [19].

## Objective

Noise in medical decision making is currently an understudied phenomenon and literature remains limited. More specifically, noise is simply termed ‘random variability’, ‘inter-rater reliability’, or ‘intra-rater reliability’ among other terms and is not clearly identified. This scoping review is one of the first which serves to label noise and bridge the knowledge gap surrounding this phenomenon in medical decision making. The objective of this scoping review is to describe noise as a feature of medical decision making, explore potential sources of noise and identify opportunities for further study.

## Methods

This review follows the Preferred Reporting Items for Systematic reviews (PRISMA) guidelines [18].

### Search strategy

A comprehensive search was carried out in PubMed in late 2023. The focus area of this review was medical decision making. PubMed was therefore selected as the optimal database as it offers a broad overview of existing literature and is a comprehensive biomedical database compared to most others. The following search strategy was applied: (‘noise’ OR ‘random variability’) AND (‘medical decision making’). Full-text screening was completed independently by two authors (KD and RL) using Covidence.

### Inclusion criteria

- Language: English or French.
- Population: Physicians, surgeons.
- Intervention/ Exposure: Medical decision making.
- Outcome: *Primary*: Qualitative or quantitative characterization of noise in medical decision making. *Secondary*: Assessment of the effect of noise on patient outcomes.
- Study design: Observational studies, longitudinal studies, randomized control trials.

### Exclusion criteria

- Language: Languages other than English or French.
- Population: Other non- MD health professionals (i.e., nurses, dentists, physiotherapists), patients.
- Intervention/ Exposure: Non-medical decision making, decision making using machine learning and data mining based tools (i.e., predictive models), shared patient- physician decision making.
- Study design: Opinion pieces, conference proceedings and abstracts, editorials, letters, and replies. Systematic reviews and meta-analyses were excluded after reference mining for additional studies that met inclusion criteria.

### Quality of evidence

Table 1 summarizes the level of evidence, found in each of the studies included in this review. The definitions for each of the levels of evidence were described by Gulpinar et al., 2014 and are defined as follows: Level I, evidence from systematic review; Level II, evidence from randomized controlled studies; Level III, evidence from non-randomized experimental studies (i.e. controlled pre-, and post-test intervention studies) or comparative studies with concurrent control groups (observational studies) (i.e. cohort studies, case-control studies); Level IV, evidence from qualitative studies (i.e., questionnaires, surveys), case series, or commentaries [12]. Level I evidence can be considered weakest, while level II and III evidence can be considered strongest.

**Table 1:** Clinical studies and type of noise identified

Clinical Study	Type of Noise	Location of Practice and Clinical Environment	Type of Data Collection and Findings	Quality of Evidence
Ebell et al., [9]	Pattern noise - Stable pattern noise	USA Family medicine, Internal medicine	Observational data collection. Physicians’ estimates of survival may be unrelated to the outcomes of the patient.	IV
Figon et al., [11]	Pattern noise - Stable pattern noise	France Family medicine	Survey data collection. Physicians may be unable to differentiate between pertinent information and background noise.	IV
Peace et al., [19]	Level noise Pattern noise - Occasion noise Pattern noise - Stable pattern noise	USA, UK Cardiology	Observational data collection. The varied competence of decision makers can contribute to noise in medical decision making. Stress, time pressure, and fatigue can all contribute to noise in decision making. Fear of making a type I or type II error can also contribute to noise.	IV
Cornou et al., 2010	Level noise	France Urology	Retrospective, quantitative data collection. Faced with the same patient characteristics, different urologists made different judgements about initiating or changing medication for a patient.	III
Williamson et al., [24]	Level noise	USA Neurosurgery	Survey data collection. Neurosurgeon decision making in traumatic brain injury (TBI) was found to be highly variable, even in the presence of evidence- based prognostic estimates.	IV
Rutkow et al., [22]	System noise Level noise Pattern noise - Occasion noise	USA General surgery, Gynecology, Otorhinolaryngology, Ophthalmology, Urology	Survey data collection. From one surgeon to the next, for some common surgical situations, the opinions provided differed to a major degree. A surgeon’s judgment with regard to the same surgical situation also differed over time.	IV
Roy et al., [21]	Level noise	Canada Neurosurgery	Survey data collection. In a group of homogeneous physicians there were significantly diverging opinions regarding the management of cerebral aneurysms.	IV
Pollack et al., [20]	System noise	USA Pediatric intensive care	Survey data collection. Higher severity- adjusted mortality in teaching hospitals may be explained by the presence of residents caring for patients in the ICU.	IV
Chamberlain et al., [4]	System noise	USA Emergency medicine	Observational data collection. Emergency departments with residents, such as those at teaching hospitals, are less effective in deciding which pediatric patients require hospital admission.	III
Divard et al., [7]	Level noise	France, USA Nephrology, Transplant surgery	Electronic Health Record data collection. While an individual physician may occasionally predict the risk of long- term allograft failure in a patient correctly, other physicians are unlikely to have the same accuracy.	III
Cozmuta et al., [6]	System noise Level noise Pattern noise - Stable pattern noise	USA Rheumatology	Survey data collection. Physicians greater than 56 years of age were more heavily influenced by the risks of all infection- related adverse events compared to their younger counterparts.	IV
Elstein et al., [10]	Pattern noise - Stable pattern noise	USA Critical care medicine	Questionnaire data collection. Some physicians were found to order many interventions, while others ordered fewer, regardless of the content of the case, the prognostic estimate given, or the physician’s estimate of the gain to the patient from being treated.	IV
McKinlay et al., [15]	Level noise	USA Oncology, Surgery	Observational data collection. Surgeons were more certain of their breast cancer diagnoses compared with nonsurgeons and were found to be less likely to order radiologic tests or a tissue sample for metastatic evaluation than were nonsurgeons.	IV
Murji et al., [17]	Pattern noise - Occasion noise	Canada Obstetrics and gynecology	Observational data collection. Obstetrics and gynecology residents’ ability to make sound clinical patient-care decisions was hindered when distractions were present when operating, with 63% of residents making at least 1 unsafe clinical decision while operating.	II

**Table 2:** Descriptive summary of noise categories as defined by Kahneman et al., [13]

Type of Noise	Definition
System noise	Variability in judgements of the same case across an institution. Composed of level and pattern noise.
Level noise	Variability in the average level of judgments by different judges. The overall patterns of each judge compared to the overall patterns of all judges.
Pattern noise	Variability that reflects a complex pattern in the attitudes of individuals to particular cases. Judge x case interaction.
Stable pattern noise	A type of pattern noise. Variability that is repeatedly observed.
Occasion noise	A type of pattern noise. Variability that is due to transient effects.

It is important to note that none of the primary studies identified for inclusion, discussed the concept of noise directly adding additional value to the results of this scoping review. We therefore extended our review to characterize the noise described in medical decision making in each study matching the description to classic definitions of noise in Table 2, as defined by Kahneman et al., [13]. When making judgements about the potential types of noise in the manuscripts reviewed, two coders (KD and RL) were involved to ensure reliability and limit risk of bias.

## Results

**Fig. 2:**
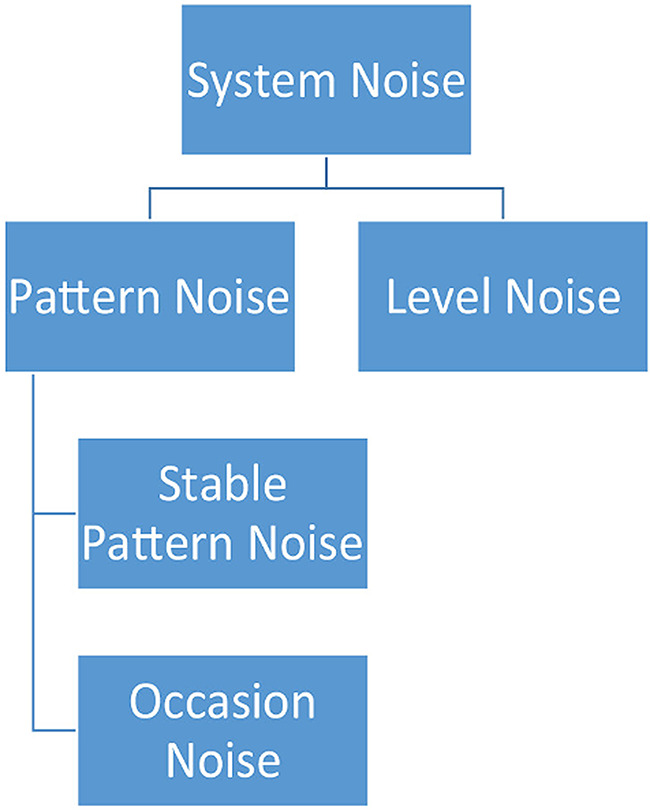
PRISMA flowchart

The search generated 2,082 results. 3 duplicates were removed for a total of 2,079 which underwent title and abstract screening. 2,046 studies were excluded at the title and abstract level. 33 studies underwent full text review of which 22 were excluded (Fig. 2), identifying a lack of literature investigating noise in medical decision making. 11 studies from PubMed and 3 studies obtained through reference mining were included. PubMed was used as the focus was decision making in medicine.

The clinical environments in which the studies included in this review were conducted, were very diverse including: family medicine, internal medicine, cardiology, urology, neurosurgery, general surgery, obstetrics/ gynecology, otorhinolaryngology, ophthalmology, pediatric and adult intensive care, nephrology, transplant surgery, emergency medicine, rheumatology, and oncology.

We determined that decision making in 7 of the 14 studies was associated with pattern noise, of which 3 studies demonstrated occasion noise, and 5 demonstrated stable pattern noise. The decision making in 8 of the included studies demonstrated level noise, and lastly the decision making in 4 of the studies demonstrated system noise, a combination of both pattern and level noise.

Peace et al., explored decision making noise when interpreting the electrocardiogram in the context of cardiac catheterization lab activation and identified several factors that contribute to noise in medical decision making as discussed in the results [19].

### Intra/ Inter rater reliability

The varied competence of different decision makers can contribute to noise in medical decision making, including intra and inter rater reliability [19]. Faced with the same patient characteristics different urologists make different judgements about whether patients with lower urinary tract symptoms should initiate or change a medication, and major variations across countries in Europe were reported for prescriptions related to benign prostatic hyperplasia (BPH) despite similarity in the prevalence and guidelines of the disease [5]. For instance, phytotherapy prescription was country specific and varied from 0 to 40% of prescriptions [5].

Similarly, neurosurgeon decision making in severe traumatic brain injury (TBI) was found to be highly variable, even in the presence of evidence- based prognostic estimates [24], suggesting low inter-rater reliability. Specifically, neurosurgeon prognostic beliefs of 6- month functional recovery were variable in both control (median 20%, IQR: 10–40%) and intervention (30%, IQR: 10-50%)[24]. Furthermore, surgeons were less likely to recommend non- surgical management when they believed the prognosis to be favorable (odds ratio [OR] per percentage point increase in 6-month functional recovery: 0.97, 95% confidence interval [CI]: 0.95–0.99) [24].

Furthermore, a study on surgical decision making and the reproducibility of clinical judgment by Rutkow et al., [22], demonstrated that for some common surgical situations, the opinions provided differed to a major degree from one surgeon to the next, and that a surgeon’s judgment with regard to the same surgical situation differed over time [22]. For instance, of 19 test cases, responses to only 2 cases showed less than a 10% reversal, responses to 5 test cases demonstrated a 10–19% chance [22]. Responses to 8 cases showed 20–29% change, responses to 3 cases showed 30-39% change, and one case showed over 40% response change suggestive of intraobserver variation [22]. Additionally, responses to the test cases showed a divergence of opinion surrounding the need for surgery [22]. Of the 19 test cases, responses to only 5 cases showed greater than 90% agreement among the respondents [22]. 3 cases demonstrated 80–89% response agreement, 3 cases showed 70-79% response agreement, 6 cases showed 60-69% agreement, and 2 showed 50-59% response agreement [22]. This was compared to control cases, where in 8 of the 9 control cases there was greater than 95% response agreement [22]. In a study by Roy et al., [21], there were significantly diverging opinions regarding the management of cerebral aneurysms even in a relatively homogeneous group of physicians, where for at least one third of the cases at least 10% of respondents opted for a decision opposite to the one of the majority.

Once again, in specialized care units, such as pediatric intensive care units (ICUs) for instance, the hospital teaching status is associated with patient outcomes [20]. A study performed in 2006 examined the association of emergency department care factors with admission and discharge decisions for pediatric patients [4]. Their findings suggest that emergency departments with residents, such as those at teaching hospitals, are less effective in deciding which pediatric patients require hospital admission [4]. Furthermore, pediatric residents were also found to be associated with higher severity- adjusted mortality rates [20]. While there have been some studies that have shown improved care in teaching hospitals compared to non teaching hospitals, an exception to this finding included pediatric intensive care [20]. In line with the above mentioned findings, it would be worthwhile exploring how intra and inter reliability change with respect to the level of training a physician has. For instance residents may show lower intra-rater reliability indicative of less consistency in their judgements compared to those with more experience. They may also show lower inter-rater reliability, in other words lower levels of agreement between two individuals.

Another study which sought to evaluate the ability of physicians to predict the risk of long- term allograft failure suggests that even if one physician may occasionally predict the risk of a patient correctly, other physicians are unlikely to have the same accuracy [7]. This can be tied back to the problem of low inter-rater reliability and can contribute to heterogeneity of practices for the same patient across physicians, including potentially highly invasive examinations, as well as unnecessary treatments that do not benefit the patient [7].

### Order of information

The order that information is presented in can also create noise and we know based on the ‘serial position effect’ [25] that the order of items in a list influences what we store in our working memory; for instance the primacy and recency effects suggest we are more likely to remember items that appeared first and last in a list compared to those in the middle [19]. The order of information flow may be another source of noise in medical decision making.

### Stress, time pressure, and fatigue

Stress, time pressure, and fatigue can all be contributors that add to noise in the decision making process. Reading information or diagnostic tests during critical scenarios and time points can contribute to noise [19]. Stress can contribute to tunnel vision and affect the ability of an individual to recognize and accurately process all the relevant information at hand [19]. Other factors to consider include the amount of time that has lapsed on an individual’s working shift and whether this affects their ability to read diagnostic images, make the appropriate referrals, and make accurate decisions [19].

### Decision fatigue

‘Decision fatigue’ is when an individual’s ability to make decisions is impaired because of the number of decisions that the individual has already had to make [19].

### Fear of making type I or type II errors

An individual’s personality as well as the environment in which they work can also contribute to noise. For example, is there more fear in a given scenario about making a type I error, such as missing a positive case, or a type II error, such as making too many false referrals [19]?. Type I error being a false positive where a null hypothesis is incorrectly rejected and a type II error being a false negative where a null hypothesis is incorrectly retained. The personalities of individual members of the medical team may play a role in decision making and the unique relationships between these personalities could influence decisions as well [19].

A study by Cozmuta et al., [6] from 2014, which examined the variability of the impact of adverse events on physicians’ decision making, found differences in age groups, where physicians greater than 56 years of age were more heavily influenced by the risks of all infection- related adverse events compared to their younger counterparts [6]. Their findings suggest that physicians differ substantially in their perception of the importance of specific adverse events [6]. Like the fear of making a type I or type II error, the perception of the importance of specific adverse events also affects a physician’s judgment - for instance whether they decide to refer a patient for additional tests or imaging. These findings can help explain the observed variability found in physicians’ day-to-day recommendations.

A study by Elstein et al., [10], assessed the effects of decision style on decision making in critical care and found a modest effect due to practice style [10]. Some physicians were found to order many interventions, while others ordered fewer, regardless of the content of the case, the prognostic estimate given, or the physician’s estimate of the gain to the patient from being treated [10]. This variation or noise in practice could be due to the hospital culture in which the physicians practice, linking back to the problem concerning physicians’ fear of making a type I or type II error [10].

## Discussion

We determined that pattern noise and level noise predominated in the studies included in this review. Of pattern noise, stable pattern noise was primarily present, followed by occasion noise. System noise was also found to be present. Possible contributors to noise include intra/ inter rater reliability, order of information, decision fatigue, and fear of making type I or type II errors among others. Previous literature has not thoroughly broken-down noise into its subtypes in the medical discipline.

### An example of noise from the public sector

While the current literature on noise in medical decision making remains limited, the phenomenon is beginning to be introduced in other fields. For instance, a study by Belle et al., 2023, looked at the management of bias and noise in the public sector using experimental evidence from healthcare [1]. Their findings suggest experts tend to make choices that are influenced by past behavior, confirmation bias, loss aversion, and equivalence framing among others [1]. Studying noise in decisions impacts crucial dimensions that are essential for effective medical service management.

Medical professionals are unaware of what others may think when making a decision, and often assume that theirs is the best judgment, however where there is judgment, there is noise. In highly complex scenarios there are many subjective components that contribute to a large degree of variability in judgements [3]. For instance, physicians’ estimates of survival can be inaccurate, and predictions can be unrelated to patient outcomes [9]. Physicians may also not be optimally trained to identify the most salient information for selecting the best treatments [11]. Through this review we highlight the importance and value of beginning to identify and accurately term noise in medicine.

### How clinical role relates to noise

Studies suggest that a clinical role may have an effect on decision making. A study on physician variability and uncertainty in the management of breast cancer showed that surgeons were more certain of their diagnoses compared with nonsurgeons [15]. Following this logic, they were found to be less likely to order radiologic tests or a tissue sample for metastatic evaluation than were nonsurgeons [15]. Tests requested were most often invasive (i.e., fine needle aspiration) or radiologic (breast ultrasound or mammogram) and therefore variables were developed to indicate whether either of these types of tests were requested [15].

Furthermore, a randomized crossover study of obstetrics and gynecology residents evaluating the effect of distractions in the operating room on clinical decision-making and patient safety found that distractions, such as pager distractions in the operating room, had an impact on both surgical and medical care that residents provided [17]. Residents’ ability to successfully complete the required laparoscopic task in the allotted time was hindered and there was a lack of accuracy in responding to clinical questions about ward patients [17]. 63% of surgical residents made at least 1 unsafe decision while operating [17]. Future studies should explore how high volumes of information, as well as competing distractions affect noise. Taking this idea a step further, it would be worthwhile to explore the effect that clinical role, and years of experience, have on the noisiness of medical decision making.

### Mitigation strategies

Algorithms are becoming increasingly important in medicine. When we engage in predictive judgments we often fail to recognize that we are operating with information that is imperfect and underestimate our objective ignorance [16]. Algorithms provide us with a framework that can negotiate these pitfalls by providing a set of rules so that a defined process can be followed instead [16].

Guidelines can also be employed to aid with diagnosis and treatment. For instance, the ‘Getting It Right First Time’ National Health Service (NHS) orthopedic project identified significant variability in orthopedic practices and proposed the use of national data registries and clear guidelines on practice in order to mitigate the unwanted variability [16]. Following this, there were substantial improvements including reduced lengths of stay and fewer inappropriate surgeries among others [16].

Furthermore, second opinions are also commonly sought in medicine. When they are reached independently, they can validate or cast doubt on a given diagnosis in question [16]. This can not only help identify noise decision making but it can also improve precision [16]. Aggregation independent decisions reduce the average error of these decisions and is known as the ‘wisdom of crowds’ [16].

### Strengths

This is the first review of its sort in medicine aiming to identify unwanted, random variability as noise across a variety of medical specialties. None of the primary studies identified for inclusion discussed noise directly. Our review therefore extends further to characterize the variability described in medical decision making in each study, matching the description to classic definitions of noise defined by Kahneman et al., [13], adding great value to this scoping review. The findings from this review are highly generalizable across a variety of medical fields as our review was able to include a large variety of specialties including family medicine, internal medicine, cardiology, urology, neurosurgery, general surgery, obstetrics/ gynecology, otorhinolaryngology, ophthalmology, pediatric and adult intensive care, nephrology, transplant surgery, emergency medicine, rheumatology, and oncology.

### Limitations

Only PubMed was used for the purpose of this review and therefore other relevant studies may have been missed. Furthermore, reviews do not offer an evidence- based synthesis for focused questions nor are they able to provide definitive guideline statements. Furthermore, a third coder could have been considered when classifying each study to the type of noise. Additionally, other definitions of each of the types of noise could have been compared to the definitions by Kahneman et al., [13], used for the purpose of coding the studies in this review. Finally, many of the studies included in the review have publication dates from over 10 years ago due to a lack of literature surrounding the topic and therefore some of the information may be outdated.

## Conclusions

Medical environments are noisy decision-making environments, and some care scenarios are noisier than others. This review is one of the first to focus on noise as a concept in medical decision making and is the first to identify the types of noise present across a variety of medical environments. Medical environments could make use of ‘noise audits’ to bring awareness to this issue. An audit can serve as a useful tool to assess the degree of variability in judgment between individuals involved in medical decision making. Future studies should focus on two main areas. The first area of focus should be on the validity of noise audits in assessing the noise present in physicians’ medical decision making. The second area of focus should involve exploring mitigating strategies that can serve as management pathways to reducing noise and improving patient care.

## Data Availability

No datasets were generated or analysed during the current study.
